# Prospective One Year Follow Up of HIV Infected Women Screened for Cervical Cancer Using Visual Inspection with Acetic Acid, Cytology and Human Papillomavirus Testing in Johannesburg South Africa

**DOI:** 10.1371/journal.pone.0144905

**Published:** 2016-01-05

**Authors:** Cynthia Firnhaber, Bridgette Goeieman, Mark Faesen, Simon Levin, Sophie Williams, Sibongile Rameotshela, Avril Swarts, Pam Michelow, Tanvier Omar, Anna-Lise Williamson, Bruce Allan, Kate Schnippel, Jennifer S. Smith

**Affiliations:** 1 Clinical HIV Research Unit, Faculty of Health Sciences, Department of Internal Medicine, University Witwatersrand, Johannesburg, South Africa; 2 Right to Care, Johannesburg, South Africa; 3 Department of OB/GYN, Coronation Hospital, University of Witwatersrand, Johannesburg, South Africa; 4 Cytology Unit, Department of Anatomical Pathology, Faculty of Health Science, University of Witwatersrand, Johannesburg, South Africa; 5 National Health Laboratory Service, Johannesburg, South Africa; 6 Institute of Infectious Disease and Division of Medical Virology, Department of Clinical Laboratory Sciences, University of Cape Town, Cape Town, South Africa; 7 National Health Laboratory Service, Groote Schuur Hospital, Cape Town, South Africa; 8 Department of Epidemiology, Gillings School of Global Public Health, University of North Carolina, Chapel Hill, North Carolina, United States of America; 9 Lineberger Comprehensive Cancer Center, University of North Carolina, Chapel Hill, North Carolina, United States of America; Istituto Nazionale Tumori, ITALY

## Abstract

**Background:**

Cervical cancer is the most common cancer in Sub-Saharan Africa. There are little of HIV-infected women one-year after screening using visual inspection with acetic acid (VIA), HPV or cytology in sub-Saharan Africa.

**Methods:**

HIV-infected women in Johannesburg South Africa were screened one year later by Pap smear, VIA and human papillomavirus (HPV) testing. Women qualified for the 12 month follow-up visit if they had a negative or cervical intra-epithelial neoplasia (CIN) 1 results at the baseline visit. Modified Poisson regression was used to analyse associations between patient baseline characteristics and progression.

**Results:**

A total of 688 of 1,202 enrolled at baseline study who were CIN-2+ negative and qualified for a 12 month follow-up visit. Progression to CIN-2+ was higher in women with positive VIA results (12.6%; 24/191) than those VIA-negative (4.4%; 19/432). HPV-positive women at baseline were more likely to progress to CIN-2+ (12.3%; 36/293) than those HPV-negative (2.1%; 7/329). Cytology-positive women at baseline were more likely to progress to CIN-2+ (9.6%; 37/384) than cytology-negative women (2.5%; 6/237). Approximately 10% (10.4%; 39/376) of women with CIN 1 at baseline progressed to CIN 2+. Women who were VIA or HPV positive at baseline were more likely to progress aIRR 1.85, CI 95% (1.46 to 2.36), aIRR 1.41 CI 95% (1.14 to 1.75) respectively.

**Conclusion:**

Progression to CIN-2+ in HIV-infected women is significant when measured by baseline positive VIA, HPV or Pap and yearly screening by any method should be considered in this population if possible.

## Introduction

Cervical Cancer, in a vast majority of cases, is preventable with adequate screening and early treatment of dysplasia. Effective cytology-based screening programs in North American, Europe and Australia/New Zealand have reduced the incidence of this cancer to <10/100,000. In contrast, in Africa where there are inadequate screening programmes, the incidence is above 30/100,000 women [1]. Cervical cancer is the most common cancer and the most common cause of cancer death in women in Sub-Saharan Africa [1]. The reason for this high prevalence is multifactorial. Inadequate access to cervical cancer screening and treatment program due to lack of skilled specialists (i.e. pathologists, gynacologists), clinic infrastructure, and transport for women to visit the clinic are a few of the reasons why appropriate screening programs have not been successfully implement in sub-Saharan Africa.

Another important reason for the high prevalence of cervical cancer is the HIV epidemic in sub-Saharan Africa. HIV-infected women have more persistent HPV infections, are more likely to be infected with multiple HPV types and are more likely to have high-grade cervical disease as compared with HIV-negative women [2]. HIV-infected women in this region are 3 to 5 times more likely to develop cervical cancer [3, 4]. Beginning in 1993, cervical cancer became one of three AIDS defining cancers per the Centers for Disease Control’s AIDS defining illnesses [5]. The World Health Organization classified cervical cancer as stage 4 AIDS defining illness in 2005 [6].

As women live longer due to widespread roll-out of antiretroviral therapy (ART), preventing other opportunistic infections, the risk of developing cervical cancer may actually increase in these countries. At present conventional Pap smears is the standard of care cervical cancer screening in South Africa but coverage is limited in many areas. Adequate implementation and access to cervical screening and treatment programs is therefore essential to maintain the health improvements achieved with ART.

Visual inspection of the cervix with acetic acid (VIA) in HIV-infected women has been shown to have a similar sensitivity for CIN-2+ to cervical cytology, albeit with lower specificity [7–9]. HPV testing has been found to have the higher sensitivity for CIN-2+, with relatively lower specificity. The World Health Organization (WHO) in the recent (December 2014) cervical cancer guidelines states there is a research gap examining the screening and follow up in HIV-infected women in resource-limited countries. It is acknowledged that, for HIV-infected populations, guidelines are based largely on expert opinion as there is insufficient scientific evidence regarding how often to screen in HIV-infected women, or comparing different screening modalities [10].

We present 1 year findings for a cervical screening study conducted among HIV-infected women in Johannesburg, South Africa. The parent study is described in detail elsewhere [7]. The objective of the current sub-study is to present the one year follow up in HIV–infected women who had negative or CIN 1+ on either Pap smear or colposcopic biopsy at the baseline visit. Three cervical screening tests were compared: VIA, cytology, and HPV DNA testing.

## Methods

### Ethics approvals

The study protocol and consent were reviewed and approved by the Human Ethics Committee (Medical) of the University of the Witwatersrand, by the University of Cape Town for HPV testing, and by the University of North Carolina for secondary data analyses.

### Study design

Women were educated in regards to the study and signed a written consent according to South African Good Clinical Practice and University of Witwatersrand ethics committee. Participants were enrolled in a prospective screening observational cohort study [7] from a Johannesburg HIV treatment clinic located in a tertiary teaching hospital. Each woman at the baseline visit was screened with a Pap smear, HPV and VIA. All women with ASCUS+ or a positive VIA had a colposcopic biopsy. In addition, every fourth participant who had both a negative Pap smear and VIA had a colposcopic biopsy at the baseline visit. Women with CIN 2+ on biopsy were referred for treatment by Loop electrical excision procedure. The other women were followed-up one year later in a sub study, if they were willing to participate and met the following criteria: qualified and participated in the baseline study visit, not pregnant, had a negative or CIN 1 histology on colposcopic biopsy or had a negative Pap smear and VIA and did not received a verification biopsy. Women who presented with sexual transmitted infections (STI) or menses were asked to return after completion of STI treatment or upon resolution of menses. A follow-up questionnaire at the one year follow-up visit was obtained through participant interviews to update status on socio-demographic characteristics, changes in medical history, ART regimen, reproductive and menstrual characteristics, and other lifestyle factors, including smoking. For further recruitment, enrolment criteria and study methodology see the parent study [7].

### Study-related procedures

Each woman at the one year follow up visit was screened using the same three screening methods as in the baseline visit: HPV Hybrid Capture 2 DNA test (QIAGEN GmbH, Hilden, Germany), conventional Pap smear cytology, and VIA, all performed using the techniques previously described[7]. Similar to the baseline visit, HPV DNA specimens were collected by the clinician and test results were not used for clinical management. The Pap smear, HPV test and VIA were all performed by study nurses. All laboratory personnel were blinded to VIA results; the HPV laboratory team was also blinded to the cytology results. Conventional Pap smears results were analyzed at the National Health Laboratory Services (NHLS) cytology unit according to Bethesda 2001 guidelines [11]; liquid based cytology is not available in the South African public sector.

After Pap smear sampling, VIA was performed, by applying 5% acetic acid to the cervix followed by a three-minute waiting period. Nurses were trained at a two-week course in Lusaka, Zambia [12]. VIA was interpreted real time by the study nurse according to the International Agency for Research on Cancer guidelines [13]. Digital images were taken using a commercially available digital camera for physician review. The final ‘VIA ‘reading used in the analysis was the reading done after review by the doctors using the digital camera images at a weekly quality assurance meeting. A colposcopic-directed biopsy was taken by study doctors for histological confirmation by an anatomical pathologist for all women with any abnormal Pap smear (ASCUS+) or a positive VIA. Lesions were biopsied and often greater than one biopsy was taken. If there were no lesions on colposcopy, biopsies were taken at the location of clock position 6 and 12 on the cervix. The study cytopathologist and anatomical pathologist were blinded to the VIA, HPV and other study results.

### Quality Assurance

For quality assurance (QA) of the VIA technique, the study gynecologist and a medical officer trained in colposcopy reviewed each digital picture and the initial VIA diagnosis of the nurse within two weeks of the VIA procedure. The medical staff reviewing the pictures were blinded to the Pap and HPV results at the time of the image review.

The cytology unit undergoes several accreditation processes from the South African National Accreditation System (SANAS) and undergoes regular proficiency testing by the Royal College of Pathologist of Australasia Quality Assurance Programme (RCPA). The cytology unit also undergoes several internal quality assurance procedures. Discrepant results between cytology and histology resulted in a review of the Pap smear slide. If discrepancy was confirmed, then a repeat colposcopic biopsy was conducted, if clinically indicated.

HPV testing QA was done per recommendation on the manufacture’s guidelines.

### Statistical methods

Subjects who were followed at 12 months were compared with those who were not in terms of demographic and clinical characteristics, {i.e. age (<30, 30–49, 50+ years), parity, CD4 count (<250, ≥ 250 cells/mm^3^), HIV viral load (<40, ≥40 copies/ml) cytology] in order to ensure that there was no selection bias in the follow-up cohort. Statistical differences were assessed using the (nonparametric) Wilcoxon rank-sum test for the continuous variables and the chi-square test for categorical ones.

At 12-month follow-up, VIA, HPV, cytology and histological biopsy results (for ASCUS+ Pap smear results) were analyzed, stratified by baseline histology or cytology status (negative or CIN 1). A modified Poisson regression approach [14] was used to calculate relative risk of progression according to baseline characteristics (i.e. age, CD4 count, VIA result, and HPV DNA infection status), with the progression of pathology at study follow-up. Progression was defined as CIN 1 progressing to CIN 2+ on colposcopic biopsy or a negative cytology/histology result to CIN 1 +. A model for progression was built and then adjusted for the baseline status (negative or CIN1) as a model covariate. 95% confidence intervals for the incidence rate ratios were computed using robust standard errors. All analysis was done within Stata v13.1 (College Station, TX).

## Results

**Fig 1:**
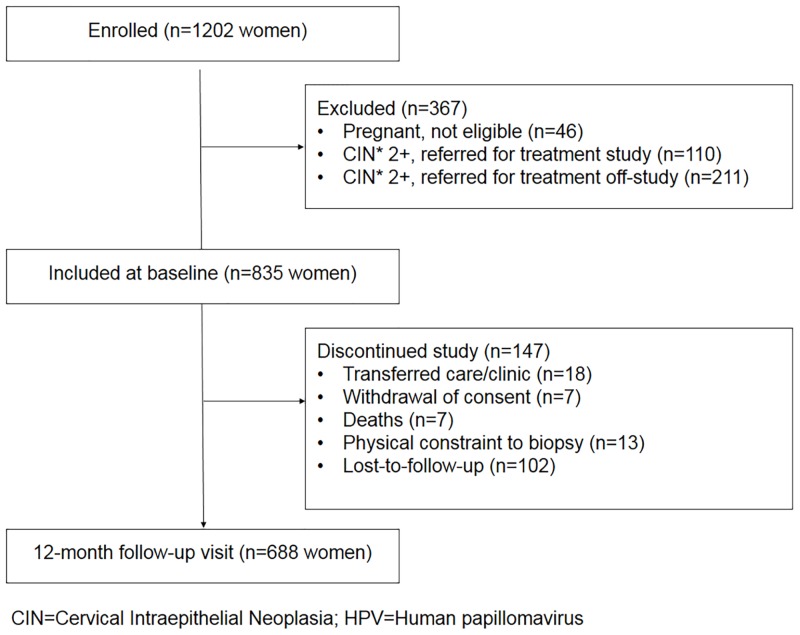
Consort diagram of participant follow-up one year later. CIN = Cervical Intraepithelial Neoplasia HPV = Human papillomavirus.

Of the 1,202 women who were initially screened, 837 women were eligible for the follow-up study. Of these women, 688 (82.2%) had a month 12 visit from March 2010 to August 2013 (Fig 1). Median age was 38 years (IQR: 33, 44). At follow-up, the median CD4 count was 490 cells/mm^3^ (IQR: 360,661) and 94.8% were on ART. Most (90.2%) of the women on ART were virally suppressed, defined as the HIV viral load measurement under 1000 copies/ml. There was no statistically significant difference in baseline demographics, HIV disease (measured by CD4 count and viral load), or reported sexual history between the women who were re-screened at 12 months and those who were not (data not shown).

Table 1 shows the results of the 12-month follow-up cervical screening tests, stratified by the baseline results from the same screening method. Of the 478 women who were VIA negative at baseline, 92 (19.2%) progressed to a positive VIA at the 12-month follow-up visit. Of the 210 women who were VIA positive at baseline who qualified for a follow-up visit (negative or CIN-1 histology), 78 (37.1%) were VIA positive (persistent) and 132 (62.9%) were VIA negative at 12 months. Of the 358 women who were negative for HPV DNA infection at baseline, 58 (16.2%) had acquired HPV infection by the time of the follow-up visit. Of the 322 women who were HPV DNA positive at baseline and eligible for the 12-month follow-up, 155 (48.1%) were HPV DNA negative at follow-up.

**Table 1:** 12-month follow-up results against baseline results from same screening methodology, VIA and HPV.

Screening method	Baseline results	12-month results		Totals
		Negative	Positive	
**VIA**	Baseline negative	386 (80.8%)	92 (19.2%)	478
	Baseline positive	132 (62.9%)	78 (37.1%)	210
	Total	518 (75.3%)	170 (24.7%)	688
**HPV**	Baseline negative	300 (83.8%)	58 (16.2%)	358
	Baseline positive	155 (48.1%)	167 (51.9%)	322
	Total	455 (66.9%)	225 (33.1%)	680^

^Baseline HPV missing (n = 1), month 12 HPV missing (n = 7)

Pap smear results at follow-up compared to Pap smear results at baseline are shown in Table 2. Of the 253 women who had a negative Pap smear at baseline, 21 (8.3%) had progressed to HSIL or ASC-H, 148 (58.5%) had progressed to LSIL and 5 (2.0%) to ASCUS. From baseline ASCUS and LSIL, 48 (19.6%) had progressed to HSIL or ASC-H. In total, compared to baseline Pap smear, 34.9% (238/683) of follow-up Pap smears indicated progression at the 12 months follow-up visit. Of the women who had a HSIL or ASC-H Pap smear at baseline (with either CIN 1 or negative histology), 26 (29.6%) persisted at HSIL or ASC-H cytology and 62 (70.4%) regressed LSIL, ASCUS, or normal Pap smear result.

**Table 2:** 12 month follow-up results against baseline results from same screening methodology, Pap smear.

Baseline results	12-month results				Totals
Pap smear	Negative	ASCUS	LSIL	HSIL, ASCUS-H	
Negative	79 (31.2%)	5 (2.0%)	148 (58.5%)	21 (8.3%)	253
ASCUS	1 (5.0%)	2 (10.0%)	16 (80%)	1 (5.0%)	20
LSIL	21 (6.5%)	3 (0.9%)	251 (78.0%)	47 (14.6%)	322
HSIL, ASCUS-H	7 (7.9%)	4 (4.6%)	51 (57.9%)	26 (29.6%)	88
Total	108 (15.8%)	14 (2.0%)	466 (68.2%)	95 (13.9%)	683*

* Baseline Pap smear missing (n = 2), month 12 Pap smear missing (n = 3)

Table 3 shows the colposcopic biopsy histology results (the gold standard) at 12-months compared to the baseline results from the different screening methods. Close to 90% (41/46) of the women who were histology negative at baseline had progressed at the 12-month follow-up screening: CIN 1. Among women that were CIN 1 at the baseline visit approximately 10% (39/376) progressed to CIN 2+.

**Table 3:** 12-month follow-up histology results against baseline results from different screening methods among women without CIN-2+ at baseline.

Screening method	Baseline results	12-month results histology^				Totals
		Negative	CIN1	CIN2	CIN3	
**VIA**						
	Baseline negative	122 (28.2%)	291 (67.4%)	14 (3.2%)	5 (1.2%)	432
	Baseline positive	19 (10.0%)	148 (77.5%)	19 (10.0%)	5 (2.6%)	191
	Total	141 (22.6%)	439 (70.5%)	33 (5.3%)	10 (1.6%)	623
**HPV**						
	Baseline negative	91 (27.7%)	231 (70.2%)	6 (1.8%)	1 0.3%)	329
	Baseline positive	49 (16.7%)	208 (71.0%)	27 (9.2%)	9 (3.1%)	293
	Total	140 (22.5%)	439 (70.6%)	33 (5.3%)	10 (1.6%)	622
**Pap smear**						
	Baseline negative	90 (38.0%)	141 (59.5%)	4 (1.7%)	2 (0.8%)	237
	Baseline ASCUS	1 (6.2%)	15 (93.8%)	0 (0.0%)	0 (0.0%)	16
	Baseline LSIL	40 (13.9%)	227 (78.8%)	17 (5.9%)	4 (1.4%)	288
	Baseline HSIL, ASC-H	10 (12.5%)	54 (67.5%)	12 (15.0%)	4 (5.0%)	80
	Total	141 (22.7%)	437 (70.4%)	33 (5.3%)	10 (1.6%)	621
**Histology**						
	Baseline negative	5 (10.9%)	41 (89.1%)	0 (0%)	0 (0%)	46
	Baseline CIN1^#^	26 (6.9%)	311 (82.7%)	30 (7.1%)	9 (2.1%)	376
	Total	31 (7.3%)	352 (83.4%)	30 (7.1%)	9 (2.1%)	422*

^ Negative cytology results presented if patient did not have colposcopy because Pap smear and VIA negative

^#^ Women with baseline CIN2+ were referred for further management at baseline and were not eligible for the 12-month follow-up study

* Baseline histology not available for n = 10; 12-month histology (or negative Pap smear) not available for n = 65 persons

Most of the 376 women who were CIN 1 at baseline persisted at CIN 1 (n = 311, 82.7%); 39 (10.4) progressed to CIN 2 or CIN 3 and 26 (6.9%) regressed to a negative histology result. In total, approximately 19% % (80/422) women with histology results at baseline and at 12 months, the cervical histology had progressed by the 12-month follow-up visit.

The relative risks of progression from baseline negative or CIN 1 and an adjusted risk of progression to CIN-2+ holding the baseline histology status constant are reported in Table 4. Most progression was from baseline histology negative (RR: 6.36, 95% CI: 4.63 to 8.74). Progression to CIN-2+ was more likely in women with positive VIA results (12.6%, 24/191) than those VIA-negative (4.4%, 19/432) aIRR: 1.85 (95% CI: 1.46 to 2.36). HPV-positive women at baseline were more likely to progress to CIN-2+ (12.3%, 36/293) than those HPV-negative (2.1%, 7/329) aIRR: 1.41 (95% CI: 1.14 to 1.75). Baseline CD4 count less than or equal to 250 cells/ mm^3^ or baseline HIV viral load undetectable (<40 copies) were not associated with progression in either the unadjusted or adjusted models. Having an STI at baseline screening was also not associated with progression. Compared to women aged 30–49 years old, women younger than 30 were at higher risk of progression (aIRR: 1.42, 95% CI: 1.01 to 2.00) and women who were 50 years or older were less likely to experience progression (aIRR: 0.50, 95% CI: 0.29 to 0.86), once the model was adjusted for baseline histology status.

**Table 4:** Relative risk of progression (from baseline negative cytology or histology to CIN 1 or from baseline CIN 1 to CIN 2+) at 12 months.

Descriptor	Value	IRR^#^	CI 95%	Adjusted IRR^	CI 95%
All women	n = 623	0.27	0.24 to 0.31		
**Baseline histology**	Negative	**6.36**	**4.63 to 8.74**		
	CIN1	REF			
**Baseline VIA**	Negative	REF		REF	
	Positive	0.77	0.57 to 1.06	**1.85**	**1.46 to 2.36**
**Baseline HPV**	Negative	REF		REF	
	Positive	0.82	0.63 to 1.07	**1.41**	**1.14 to 1.75**
**Baseline CD4 count**	>250 cells/mm^3^	REF		REF	
	≤250 cells/mm^3^	1.01	0.35 to 1.57	1.08	0.71 to 1.64
**Baseline viral load**	Detectable**	REF		REF	
	Undetectable***	1.20	0.92 to 1.56	1.00	0.80 to 1.24
**Age category**	<30 years	0.93	0.62 to 1.39	**1.42**	**1.01 to 2.00**
	30 to 49 years	REF		REF	
	50 years +	0.56	0.30 to 1.04	**0.50**	**0.29 to 0.86**
**Sexually transmitted infection at baseline**	No STI present	REF		REF	
	STI present	0.93	0.71 to 1.21	0.99	0.79 to 1.23

^#^ Relative risk calculated using incidence rate ratios, Poisson distribution with robust standard errors.

^ Adjusted for baseline histology results.

** detectable is ≥ 40 copies/ml

***undetectable is <40 copies/ml

## Discussion/Conclusion

Previous cross-sectional and prospective studies have shown both high rates of cervical dysplasia (LSIL+) at baseline and high rates of incident disease in HIV-infected women in South Africa ranging from 34% to 75% [7,15,16]. Progression rates measured by cervical Pap smear results in HIV-infected women in South Africa have been shown to be high in two cohorts of HIV-infected women from Johannesburg. Omar et al data showed the rate of progression from negative to LSIL was 9.6/100 person years (95% CI: 8.3 to 11.1) and from LSIL to HSIL 4.6/100 py (95% CI: 3.9 to 5.5) after approximately 1-year follow-up [15]. Firnhaber et al showed progression rates of 14.6/100 py (95% CI: 11.5 to 18.5) from negative to LSIL and LSIL to HSIL of 10.8/100 py (95% CI: 8.1 to 14.4) [17]. Our current study demonstrated similar disease progression from a negative histology baseline result to CIN 1 in approximately 60% of women. Histological progression from CIN 1 to CIN 2+ was approximately 10% in the one year of follow-up.

Comparing these results to HIV-negative women seen within the same government public system in Johannesburg showed a baseline abnormal cytology of 11.4% (1.8% HSIL) with a progression rates from a negative Pap smear to cervical abnormalities of 6.5% within a year. Progression to HSIL during this time period was rare, less than 0.5% which would allow for longer intervals for screening in HIV-negative women [18].

Our data shows that if a woman is VIA positive but has normal or CIN 1 histology, her risk of progression to CIN 1+ histology within one year is nearly two times higher than the risk of progression in VIA negative women. Similarly, if a women is HPV positive but has normal or CIN1 histology, her risk of progression within one year is 40% higher than the risk for HPV negative women. Importantly, 19% (80/422) women progressed from negative or CIN 1 histology to CIN1+ histology, suggesting a treatment by cryotherapy or other method at the same visit (see and treat) may be warranted. Kuhn et al showed that HIV-infected women compared to HIV-negative women who were screened by either HPV or VIA but not treated had a high rate of CIN 2+.Of the untreated women 14.9% progressed to CIN 2+ compared to 4.6% who were treated. HIV-infected women screened with VIA and treated with cryotherapy had a drop in CIN 2+ by 7.4% and women screened by HPV and treated with cryotherapy had a 12% reduction of CIN 2+ [8].

Two studies from Johannesburg have shown that HAART in this population reduces the cervical dysplasia progression rate [17,19], but in should be noted this gain in slowing the rate of cervical disease progression may be overcome by women living longer. In this current study, the baseline cervical dysplasia prevalence were high. One year later there was significant progression, despite over 90% of women taking ARVs the majority of the women on ARVs had suppressed HIV viral loads (<1000 copies/ml) at the month 12 study visit.

Interestingly, our study showed that age over 50 years was protective of progression. This could be due to a selection bias as the women who had CIN 2+ disease were sent for treatment and not eligible for a follow-up visit at month 12.

HIV infection has been shown to increase persistence and reduce clearance of HPV infection [20,21]. At the baseline visit of this study, 731 women were HPV positive. Of the women who qualified for a month 12 visit 155 women no longer had HPV infection for a rate of HPV infection clearance of 21.2% (155/731). This is higher than the Denny et al cohort where only 6% cleared there HPV in 36 months [16]. This difference maybe as result of the women with high-grade lesions were referred for treatment and did not qualify for the 12-month screening follow up. Additionally, the Denny et al study was done in the early 2000’s when ARVs were not readily available in South Africa. In contrast, approximately 90% of the women in our study were on ARVs and virally suppressed. Good adherence to ARV medication has been shown to decrease the persistence of HPV infection in HIV-infected women [22,23].

This study was not designed to evaluate regression as women with CIN 2+ histologically were referred for treatment and did not qualify for the month 12 visit. However, the likelihood of regression from VIA positive to negative results or HPV infection clearance over the one follow up might be inferred or estimated from these study results. Women who were VIA positive at the baseline visit (n = 529) and qualified for month 12 visit, 24.8% (132/529) of these women regressed to a negative VIA result. This information and the increased risk of women who are VIA positive progressing to high-grade disease in areas with limited access to pathology, treatment for dysplasia or cervical cancer, may negate the concerns of overtreatment of HIV-infected women when using the VIA see and treat method.

Another limitation of this study is the progression may not have reflected incident disease or change of disease but prevalent disease that was missed by initial cytology or histology. This possibility was minimized by the continuing internal and external quality assurance programs by the NHLS anatomical pathology services, secondary review of discrepant results and if necessary a second biopsy.

These results provide important information required for resource allocation for cervical cancer screening, both in terms of guidelines of when to screen and also improving access to screening in the country with the largest HIV epidemic. Progression rates are high in this population and a positive VIA and HPV test demonstrated a higher risk of progression within one year. VIA is at present is being implemented in several sub-Saharan Africa countries through a variety of governmental and donor funded programs. Of the 432 women with a baseline negative VIA, 19 (4.4%) had CIN 2+ a year later. Although comparing different modalities, this is about 8 times the progression seen in the HIV-negative population (<0.5%) in Johannesburg using Pap smears indicating that a screen and treat program in HIV-infected women with negative baseline results should consider reduced screening intervals.

HIV-infected women are at significant risk for progression of their cervical dysplasia. Whilst there is some evidence that ARVs might slow progression, the disease is not eliminated in many of these women. In this cohort, progression still occurred with a relative high median CD4 count and with the majority of women with suppressed viral load. Countries need to aggressively ramp-up the scale of cervical cancer screening and frequency of screening (maybe yearly) using one of the appropriate screening methods per the capacity and resources of the country for HIV infected women. VIA is a possible option in countries with limited resources and poor access to screening. Longer term studies with information on the relative costs of screening methods are needed to better inform screening policies for the individual countries. Improved access to screening is imperative to maintain the gains of health achieved in our HIV-infected women.
